# Mendelian randomization analysis does not reveal a causal association between migraine and Meniere’s disease

**DOI:** 10.3389/fneur.2024.1367428

**Published:** 2024-05-09

**Authors:** Kangjia Zhang, Yong Zhang, Weijing Wu, Ruosha Lai

**Affiliations:** Department of Otolaryngology, Head and Neck Surgery, The second Xiangya Hospital of Central South University, Changsha, Hunan, China

**Keywords:** migraine, Meniere’s disease, Mendelian randomization, single-nucleotide polymorphisms, genetic vulnerability

## Abstract

**Background:**

According to observational research, migraine may increase the risk of Meniere’s disease (MD). The two have not, however, been proven to be causally related.

**Methods:**

Using Mendelian random (MR) analysis, we aimed to evaluate any potential causal relationship between migraine and MD. We extracted single-nucleotide polymorphisms (SNPs) from large-scale genome-wide association studies (GWAS) involving European individuals, focusing on migraine and MD. The main technique used to evaluate effect estimates was inverse-variance weighting (IVW). To assess heterogeneity and pleiotropy, sensitivity analyses were carried out using weighted median, MR-Egger, simple mode, weighted mode, and MR-PRESSO.

**Results:**

There was no discernible causative link between genetic vulnerability to MD and migraine. The migraine dose not increase the prevalence of MD in the random-effects IVW method (OR = 0.551, *P* = 0.825). The extra weighted median analysis (OR = 0.674, *P* = 0.909), MR-Egger (OR = 0.068, *P* = 0.806), Simple mode (OR = 0.170, *P* = 0.737), and Weighted mode (OR = 0.219, *P*= 0.760) all showed largely consistent results. The MD dose not increase the prevalence of migraine in the random-effects IVW method (OR = 0.999, *P* = 0.020). The extra weighted median analysis (OR = 0.999, *P* = 0.909), MR-Egger (OR = 0.999, *P* = 0.806), Simple mode (OR = 0.999, *P* = 0.737), and Weighted mode (OR = 1.000, *P* = 0.760).

**Conclusion and significance:**

This Mendelian randomization study provides casual evidence that migraine is not a risk factor for MD and MD is also not a risk factor for migraine.

## 1 Introduction

Meniere’s disease (MD) is a chronic inner ear ailment characterized by recurrent vertigo, tinnitus, sensorineural hearing loss (SNHL), and auditory fullness. MD frequency and incidence vary by ethnic and geographic background around the world, ranging from 3 to 513 per 100,000 people. The phenotype is variable and may be linked to other comorbidities, such as migraines, respiratory allergies, and autoimmune disorders, and it should be considered as a clinical syndrome with different etiologies (1). Its etiology and molecular pathophysiology are unknown. Endolymphatic hydrops(EH) has been one of the etiologic factors of MD (2). The group MD-dg (ES degeneration) and the group MD-hp (ES hypoplasia) expressed variable radiological features of the temporal bone, which indicated that the disease may have different endotypes to illustrate its phenotypes (3). Several genes have been reported in Family MD(FMD), nine autosomal dominant (FAM136A, PRKCB, COCH, DPT, SEMA3D, TECTA, GUSB, SLC6A7), four autosomal recessive (HMX2, LSAMP, OTOG, STRC) and six about digenic inheritance in MD (MYO7A, ADGRV1, CDH23, PCDH15, USH1C, SHROOM2) (4).

Migraine is a common and complex neurological disorder that involves both neuronal and vascular mechanisms. It affects about 15-18% of the general population, which can be divided into migraine with aura (MA) and without aura (MO) and is characterized by recurring episodes of several headaches, vomiting, nausea, and hypersensitivity to sound, light and smell (5). Three genes contribute to family hemiplegic migraine (FHM): calcium voltage-gated channel subunit alpha1 A—CACNA1A (FHM1); ATPase Na+/K + transporting subunit alpha 2—ATP1A2 (FHM2) and sodium voltage-gated channel alpha subunit 1—SCN1A (FHM3). Five other genes can also be linked to FHM: PRRT2, SLC2A1, PNKD, SLC1A3 and SLC4A4. In addition, genes associated with neuronal, vascular, ion channel/homeostasis, glutamatergic transmission, and nitic oxide or oxidative stress play an important role in migraine (5). EH is a common pathology shared by both MD and vestibular migraine, VM, which remains a clinical diagnosis with no highly accurate tests for the disorder (6, 7). VM may experience symptoms of MD, and most VM patients experience episodes of vertigo, which occur separately from headaches. As a result, VM patients can receive a false diagnosis of MD patients. It is frequently challenging to distinguish between these two disorders under these conditions. As mentioned above, migraine and MD have various phenotypes, often diagnosed by clinical features, showing some overlapped clinical symptoms, involving multiple etiologies and part of them can be related to several genes. Thus, more and more studies are linking migraine to recurrent vestibular problems (8, 9). A recent population-based study in Korea found a bidirectional relationship between MD and migraine, sufferers with MD were more likely to get migraine, and migraine sufferers were more likely to develop MD (10). Frank et.al. (11) even considered the possibility that the illnesses of MD, VM, and cochlear migraine (CM) are variations of the same entity known as otologic migraine and are part of a spectrum of disorders associated with central sensitivity. But whether the migraine will develop to MD or increase the risk of MD is unknown.

Mendelian randomization (MR) is a new genetic epidemiology approach that uses genetic variation as an instrumental variable (IV) for risk variables, allowing researchers to completely understand the causal effect of exposure on results (12). Because genes are assigned at random during meiosis, this method eliminates other potential confounders and interferences via reverse causality, resulting in more significant causal results than traditional observational research (13). As a result, in this investigation, we used existing, publicly available, large-scale genome-wide association studies (GWAS) to conduct a two-sample MR analysis to further clarify the causative hypothesis of migraine and its relationship with MD risk.

## 2 Methods

### 2.1 Study design

We looked into the connection between MD and migraines with a two-sample MR configuration. Three main hypotheses should form the foundation of a compelling MR design: (1) genetic variation is strongly and directly related to exposure (migraine); (2) genetic variation is unrelated to potential confounders; and (3) genetic variation influences outcome (MD) exclusively through exposure and not through other pathways (14).

### 2.2 GWAS data for migraine and MD

Genetic information related to exposure data (migraine/MD) and outcome data (MD/migraine) was obtained from publicly available GWAS abstract summary data, which are accessible through the GWAS catalog.^1^ This study’s most recently published GWAS included 484,598 participants (13,971cases and 470,627 controls) of migraine dataset (ebi-a-GCST90038646) and 482,774 participants (1,526 cases and 481,248 controls) of the MD dataset (ebi-a-GCST90018880).

### 2.3 Instrumental variable selection

To choose scientifically viable SNPs, a criterion for genetic instrument selection was proposed. The exposure was migraine SNPs (*p* < 5 × 10^–8^) and MD (*p* < 5 × 10^–6^), were chosen as instrumental factors to reach genome-wide significance. The linkage disequilibrium threshold was set to *r*^2^ = 0.001 within a distance of 10,000 kb. Finally, we computed the total F-statistic, which is *F* = beta^2^/se^2^. We chose SNPs with *F* > 10 in this process to guarantee that each SNP had enough strength for the analysis.

### 2.4 Statistical analyses

To evaluate the causal relationships between MD and migraine, we conducted random Mendelian effects analyses. Initially, we examined the effect of exposure on MD by regressing genetic variance in exposure (migraine) on the outcome (genetic variance in susceptibility to MD), with each conflict representing one data point. Utilizing an inverse variance-weighted (IVW) random-effects approach in the primary analysis with a *p*-value of <0.05. We selected this method as the main approach for this MR because it yields estimates that should be higher than the Wald ratio estimates of variance (15).

In addition, we performed several sensitivity analyses using MR-Egger, weighted medians, simple mode, and weighted mode as a complement to IVW to identify any bias in evaluating MR hypotheses. MR-Egger analyses allow for pleiotropy for all genetic variants, but the magnitude of pleiotropy (from genetic variant to outcome and bypassing exposure) should be separate from the main effect’s (from genetic variation to exposure) extent. Apart from this, we also applied the MR-Egger intercept test to detect unbalanced horizontal pleiotropy. If pleiotropy was present, then the analysis yielded a *p*-value of <0.05 for the intercept (16). The weighted median provides a robust estimate, even if up to 50% of the genetic variation violates the assumption (17). The Mendelian Randomization Pleiotropy RESidual Sum and Outlier (MR-PRESSO) method can also detect and remove outliers to obtain relatively unbiased estimates (13). For a meaningful forecast, we used Cochrane Q-values to assess heterogeneity and visualized funnel plots by plotting the inverse distribution of standard errors for each SNP around the MR estimates (16). In the leave-one-out method, each SNP was removed in turn, and the remaining SNPs were used to calculate the causal effect of gene prediction exposure (migraine) on the outcome (MD) (18).

In order to analyze whether there is an effect on migraine from MD, we did an analysis using the same method by exchanging the exposure to MD, and the outcome became migraine.

All MR analyses in this study were performed using R software (version 4.2.5) and the “TwoSampleMR” package (version 0.5.7).

## 3 Results

### 3.1 Migraine will not increase the risk of MD

**Figure 1:**
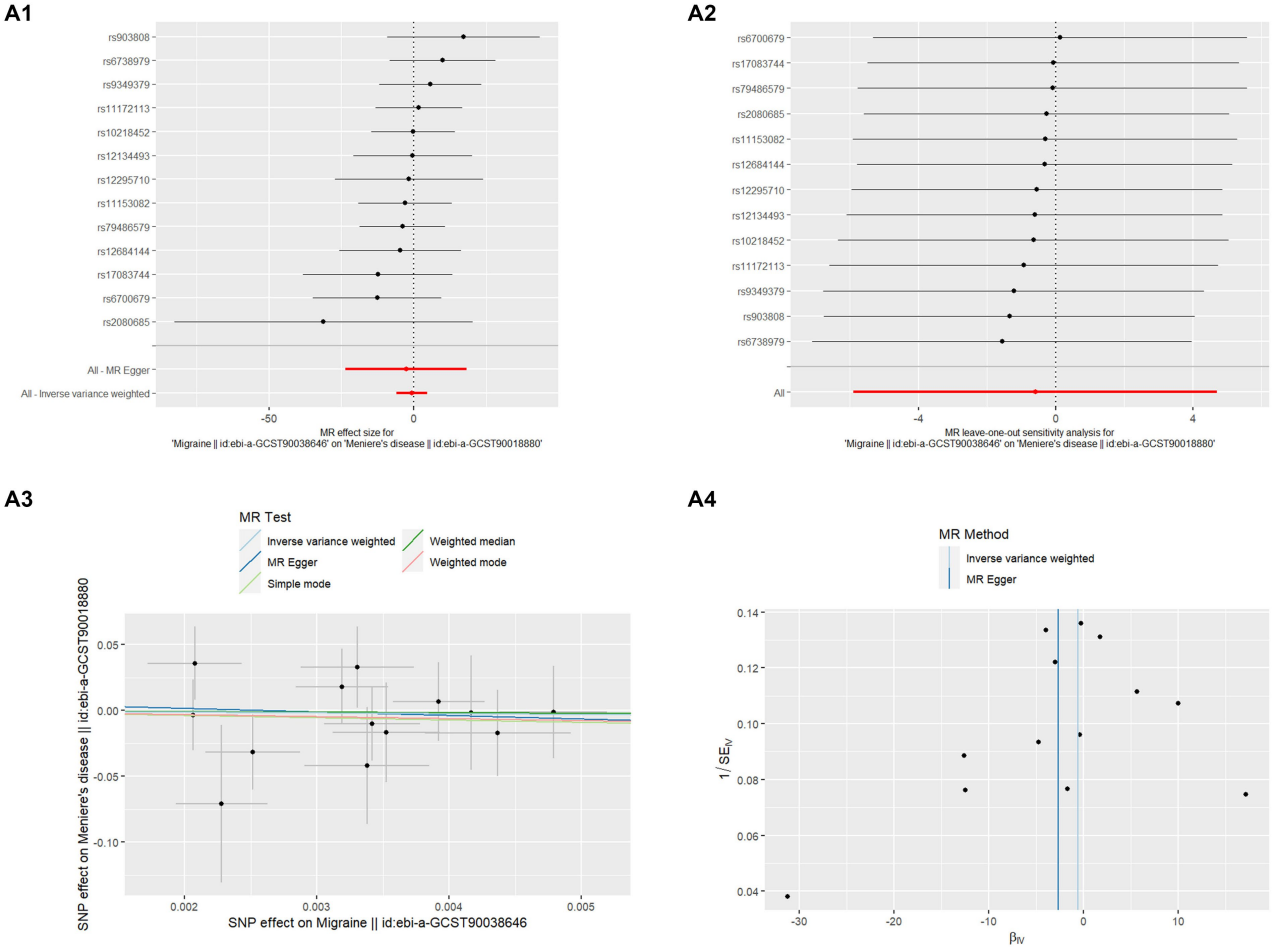
MR analysis of Migraine on MD **(A1)** Forest plot of the potential effects of migraine associated SNPs on MD. **(A2)** Leave-one-out plot of migraine on MD. **(A3)** Scatter plot of the potential effects of migraine associated SNPs on MD. **(A4)** Funnel plot of the casual effects of migraine “related SNPs on MD.

**Figure 2:**
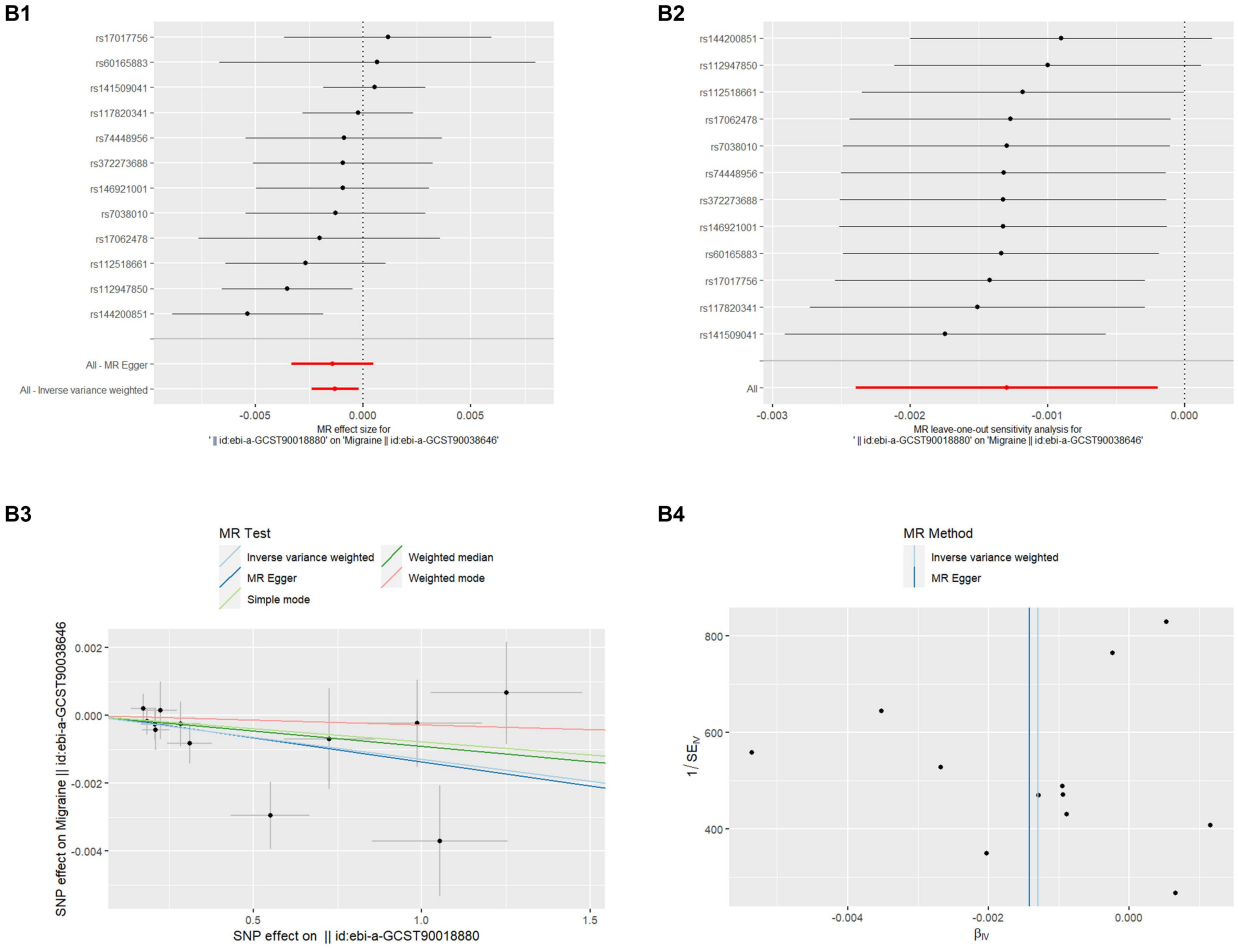
MR analysis of MD on migraine **(B1)** Forest plot of the potential effects of MD associated SNPs on migraine. **(B2)** Leave-one-out plot of MD on migraine. **(B3)** Scatter plot of the potential effects of MD associated SNPs on migraine. **(B4)** Funnel plot of the casual effects of MD related SNPs on migraine.

This study used MR to examine the relationship between migraine and MD in two samples. The comprehensive outcomes are listed in Table 1. When choosing migraine as the exposure and MD as the outcome, there was no discernible causal link between migraine and MD risk in the IVW approach, according to MR analysis employing 13 SNPs (OR = 0.551, *p* = 0.825). The extra weighted median analysis (OR = 0.674, *p* = 0.909), MR-Egger (OR = 0.068, *p* = 0.806), simple mode (OR = 0.170, *p* = 0.737), and weighted mode (OR = 0.219, *p* = 0.760) all showed largely consistent results. There was no statistically significant correlation found in scatter and forest plots between genetic predisposition to migraine and MD (Figures 1A1,A3). Additionally, the funnel plot visualization is displayed in Figure 2A4, and there was no indication of observed heterogeneity in effect estimates MR-Egger (Cochrane’s *Q* = 7.330, Q_df = 11, *p* = 0.772) and IVW (Cochrane’s *Q* = 7.371, Q_df = 12, *p* = 0.832). Furthermore, no aberrant IV was seen using the MRPRESSO technique. A single SNP may not be the cause of the link between migraine and MD, according to the findings of the leave-one-out sensitivity analysis (Figure 1A2). Crucially, directional pleiotropic effects in genetic variation were not shown by MR-Egger regression (intercept = 0.007, Se = 0.036, *p* = 0.843). This implies the robustness of our analysis.

**Table 1:** MR analyses showing the associations of genetic migraine with the risk of MD.

Exposure	Outcome	MR approaches	SNPs	OR	*p*-value
		MR Egger	13	0.068	0.806
		Weighted median	13	0.674	0.909
Migraine	MD	IVW	13	0.551	0.825
		Simple mode	13	0.170	0.737
		Weighted mode	13	0.219	0.760
		MR-PRESSO	13	/	0.842
		MR Egger	12	0.999	0.175
		Weighted median	12	0.999	0.247
MD	Migraine	IVW	12	0.999	0.020
		Simple mode	12	0.999	0.580
		Weighted mode	12	1.000	0.784
		MR-PRESSO	12	/	0.375

### 3.2 MD will not increase the risk of migraine

Even though migraine does not affect the risk of MD, the papers have reported the high prevalence of Migraine in the MD population (8) so we also analyzed whether MD affects the risk of Migraine which is also listed in Table 1. There was no discernible causal link between migraine and MD risk in the IVW approach, according to MR analysis employing 12 SNPs (OR = 0.999, *p* = 0.020). The extra weighted median analysis (OR = 0.999, *p* = 0.909), MR-Egger (OR = 0.999, *p* = 0.806), Simple mode (OR = 0.999, *p* = 0.737), and Weighted mode (OR = 1.000, *p* = 0.760) all showed largely consistent results. There was no statistically significant correlation found in scatter and forest plots between genetic predisposition to migraine and MD (Figures 2B1,B3). Additionally, the funnel plot visualization is displayed in Figure 2B4, and there was no indication of observed heterogeneity in effect estimates MR-Egger (Cochrane’s *Q* = 12.047, Q_df = 10, *p* = 0.282) and IVW (Cochrane’s *Q* = 12.078, Q_df = 11, *p* = 0.358). Furthermore, no aberrant IV was seen using the MR-PRESSO technique. A single SNP may not be the cause of the link between migraine and MD, according to the findings of the leave-one-out sensitivity analysis (Figure 2B2). Crucially, directional pleiotropic effects in genetic variation were not shown by MR-Egger regression (Intercept = 5.405, Se = 0.0003, *p*  = 0.876). This implies the robustness of our analysis.

## 4 Discussion

To the best of our knowledge, this is the first study to use two-sample MR analysis to assess the causal relationship between migraine and MD. To look into the possible causal relationship between MD and migraine, this study thoroughly analyzed the largest database, GWAS. Our results imply that there isn’t a definite cause-and-effect relationship between them

MD was first described by Prosper Meniere in 1861, he stated ‘‘. . . it is not less certain that cerebral states, called migraine, give place in the end to similar attacks, and the deafness which arises in these circumstances would seem to us inevitably to be related to a disease of the same nature (11).”

Between 1992 and 2001, there were no criteria for VM, and the diagnosis of MD was not standardized. Additionally, some studies didn’t match the age and sex in the control group, leading to the highly variable prevalence of migraine and MD that could not be trusted (19, 21).

Between 2001 and 2011, most studies found that MD is more common in female patients and people aged (40-60) years old, so studies have matched the control group by age and sex. In 2001, Neuhauser (22) proposed the criteria for the diagnosis of VM. In 1995, the American Academy of Otolaryngology-Head and Neck Surgery, AAO-HNS, criteria for MD was formed (23), but VM was not officially included in the diagnosis of migraine. Therefore, there is a lack of diagnosis of migraine with vestibular symptoms as an aura (8, 24–26).

Between 2012 and 2017, VM was included in the diagnostic criteria of the International Classification of Headache Disorders (ICHD-3) in 2012 (27). The majority of studies still rely on the 1995 diagnostic criteria for MD, despite the updated criteria in 2015 (28). Additional research has been conducted to distinguish the clinical symptoms and tests between migraine and MD. However, there are variances in the comprehension of these two conditions in otology and neurology. The analysis of possible VM (pVM) and probable or possible MD (pMD) were also not incorporated in these studies. The pMD and pVM patients can cause differences in the data results, so it is necessary to conduct a detailed classification study of the two groups of patients (28–33).

Between 2018 and 2023, based on the understanding of the overlap syndrome of VM, MD, and VMMD, it was proposed in 2017 that the three diseases need to be classified and studied. At the same time, the inclusion of population data should be more extensive. Due to the insignificant research results of different clinical diagnoses, many scholars believe that the two may be different manifestations of the same disease (10, 11).

It can be difficult to differentiate between VM and MD, as the diagnosis is primarily based on clinical criteria. Many studies have shown a significant overlap in symptoms between the two conditions (34, 35). That is why an increasing number of researchers think that MD and migraine may share a pathogenic etiology, and some even think that MD develops as a result of migraine (11, 24, 25, 29). A multitude of underlying factors, including genetics, autoimmunity, chemical exposure, viral infection, inflammation, ischemia, altered intralabyrinthine fluid dynamics, and cellular and molecular mechanisms, interact to cause MD (36, 37). The hypotheses for VM included internal auditory artery vasospasm, trigeminovascular system involvement, and a malfunction in sensory functioning at the vestibular system, thalamus, or cortical levels (38). Repetitive vascular difficulties during migraine episodes may eventually cause irreparable loss of vestibular and cochlear function. The high frequency of migraine in MD may be explained by neurotransmitters that influence vestibular function, such as dopamine, serotonin, noradrenaline, and neuropeptides, such as calcitonin gene-related peptide. Additionally, aberrant neurotransmitter release and an ion channel abnormality can also be the mechanisms of both diseases (8). A local increase of extracellular potassium causes both the spreading depression in migraine and lethal consequences on hair cells in the inner ear (34).

Despite having noticed that the association between MD and migraine has been extensively studied in the literature, the MR analysis in this study found no relationship, which could be attributable to the following factors.

We have detailed various errors that may have influenced the results of the data from different studies. In conclusion (a) the main evidence for the link between migraine and MD is the clinical observation and epidemiological finding of the coexistence of vertigo and other migraine symptoms, but there is partial error and delayed diagnosis of the two disorders (39) (b) Otologists and neurologists have different perspectives on the clinical presentation, pathophysiology, and management of VM, which will lead to statistical variations that can also affect the results (40) (c) In statistics, prevalence and incidence are used together in many literature, which can lead to confusion between the two concepts. An increase in incidence may be mistaken for an increase in prevalence, while the incidence may be due to the overlap of the two diseases and the recurrence of the disease (d)VM, though not universal, is the most common cause of recurrent spontaneous vertigo; with a lifetime prevalence of approximately 1–2.7% in the general population, it is more common than MD. However, for a significant proportion of individuals, the co-occurrence of vestibular symptoms and migraine is merely coincidental (41, 42).

For the hypothesis, although the co-occurrence of migrainous characteristics and MD may be explained by a variety of vascular mechanism changes, aberrant neurotransmitter release, and ion channel dysfunction. Firstly, there was no difference in the prevalence of vascular risk factors between delayed MD (*N* = 75, 7.6% patients) and non-delayed MD (*N* = 913, 92.4% patients) (30). Secondly, about genetics, the FMD (Family MD) subtyping indicates that there will be two forms of FMD: migraine-associated and migraine-free, which will reflect the genetic heterogeneity found in autosomal dominant FMD (30). MD-affected families are incredibly diverse. There was no commonality in terms of migraine, age at onset, expectation, or penetrance (43). Thirdly, Frejo et.al. observed that cluster 4 (SMD with migraine) has an earlier age of onset than the rest of the groups and there are significant differences in delayed MD, FMD, migraine, and autoimmune disease (AD) between the groups, it implies that relationships between various subgroups of MD and migraine will differ dramatically (31). Additionally, psychiatric comorbidity was considerably more common in patients with VM and MD (MD = 57%, VM = 65%), particularly in those with anxiety and depressive disorders. Previous research revealed that anxiety and depression are more common in MD patients than in healthy people (44). Thus, certain recurrent attacks may be mistakenly perceived as organic by both the attending physician and the patients.

For the limitations of the study, Firstly, migraine is a complex heterogeneous disorder, and patents’ lifestyle and environmental factors have a significant role in the phenotype expressivity. MD is not a single disease. It should be considered a complex syndrome with several endophenotypes according to genetic, immunological, and radiological markers. However, the MR approach cannot investigate epigenetic or environmental factors, that may have a significant influence in both conditions, such as hormone variation on brain fluid balance, effects of dietary habits on blood vessels and so on. Secondly, migraine with aura is a narrower phenotype that should be investigated separately from migraine without aura. The study didn’t analyze it solely, and we are also not investigating VM separately. Thirdly, common variants associated with migraine cannot explain a rare endophenotype in MD, such as monogenic MD or autoinflammatory MD associated with proinflammatory cytokines. All of these reasons can influence the association between the two conditions.

## 5 Conclusion and significance

Our Mendelian investigation demonstrated that there is no association between migraine and MD, even though numerous studies in the literature have examined this relationship and concluded that the two are correlated. However, there is a dearth of data regarding the various subgroups of migraine and MD. Certain subgroups of migraine, such as VM, may be significantly associated with particular subgroups of MD. Consequently, to facilitate both disease diagnosis and thorough treatment of various disease classifications, our clinical observation study must classify these two diseases in depth.

## Data availability statement

The original contributions presented in the study are included in the article/supplementary material, further inquiries can be directed to the corresponding author.

## Author contributions

KZ: Writing – original draft, Data curation, Formal analysis, Methodology, Software. YZ: Writing – review & editing, Data curation, Methodology, Software. WW: Supervision, Writing – review & editing. RL: Supervision, Writing – review & editing, Formal analysis, Funding acquisition.

## Footnotes

^1^ https://gwas.mrcieu.ac.uk/
